# 血液透析滤过处理CAR-T治疗后IL-6受体抑制剂治疗无效的3~4级细胞因子释放综合征3例

**DOI:** 10.3760/cma.j.issn.0253-2727.2022.06.009

**Published:** 2022-06

**Authors:** 诗彧 陈, 伟红 陈, 晓春 万, 新 杜, 畅如 罗, 晓清 李, 晓瀚 张

**Affiliations:** 1 深圳大学第一附属医院/深圳市第二人民医院血液内科，深圳 518035 Department of Hematology, The First Affiliated Hospital of Shenzhen University, Shenzhen Second People's Hospital, Shenzhen 518035, China; 2 中国科学院深圳先进技术研究院、生物医药与技术研究所蛋白与细胞药物研究中心，深圳 518055 Protein and Cell Drug Research Center, Institute of Biomedicine and Technology, Shenzhen Institute of Advanced Technology, Chinese Academy of Sciences, Shenzhen 518055, China

**Keywords:** 血液透析滤过, 嵌合抗原受体T细胞, 细胞因子释放综合征, IL-6受体抑制剂, Hemodiafiltration, Chimeric antigen receptor T cells, Cytokine release syndrome, IL-6 receptor inhibitor

## Abstract

**目的:**

观察血液透析滤过（HDF）处理嵌合抗原受体T细胞（CAR-T细胞）免疫疗法后IL-6受体抑制剂治疗无效细胞因子释放综合征（CRS）的有效性及安全性。

**方法:**

回顾性分析2015年7月至2021年7月深圳大学第一附属医院血液内科经CAR-T细胞治疗后出现托珠单抗治疗无效的3～4级CRS并接受HDF治疗的3例患者，包括急性B淋巴细胞白血病（B-ALL）2例，弥漫大B细胞淋巴瘤（DLBCL）1例。

**结果:**

患者在HDF治疗结束后12 h内临床症状（体温、血压、血氧）缓解，细胞因子IL-6、IL-10、TNF-α、INF-γ及C反应蛋白（CRP）明显下降。随访3个月未发现HDF治疗相关不良反应。

**结论:**

HDF控制CAR-T细胞治疗后托珠单抗治疗无效3～4级CRS安全可行。

## Abstract (en)

**Objective:**

To investigate the efficacy and safety of hemodiafiltration (HDF) in treating CAR-T related grade 3–4 cytokine release syndrome after ineffective treatment with IL-6 receptor inhibitors.

**Methods:**

Between July 2015 and July 2021, retrospective analysis of hemodiafiltration for the treatment of 3 patients, including 2 cases of acute B-lymphoblastic leukemia and 1 case of diffuse large B-cell lymphoma, with grade 3–4 CRS after CAR-T cell therapy and ineffective treatment with IL-6 receptor inhibitor was carried out.

**Results:**

The patient's clinical symptoms, including body temperature, blood pressure, and blood oxygen, were relieved within 12 hours of all treatments, and the cytokines (IL-6, IL-10, TNF-α, INF-γ) and C-reactive protein (CRP) levels decreased significantly. No adverse side effects were observed during the follow-up period of 3 months.

**Conclusion:**

HDF can be a safe and feasible method to treat CAR-T related grade 3–4 CRS after ineffective treatment with IL-6 receptor inhibitors.

细胞因子释放综合征（CRS）是指嵌合抗原受体T细胞（CAR-T细胞）在靶向杀灭肿瘤细胞的过程中T细胞及单核巨噬细胞系统活化，释放大量细胞因子，诱发患者出现全身性的炎性反应[1]。CRS是导致CAR-T治疗失败甚至死亡的重要原因[2]。目前主流应用于控制CRS的药物是糖皮质激素和IL-6受体抑制剂[3]–[4]。然而大剂量使用糖皮质激素可能会损害CAR-T细胞活性，进而影响患者的长期疗效[5]–[6]。IL-6受体抑制剂托珠单抗存在一些局限性：① 在3～4级CRS且病情进行性加重的情况下不能够快速起效；② 增加神经毒性的发生率，增加类风湿性关节炎患者发生血细胞减少和感染的风险[7]；③ 对非IL-6为主要驱动因素的患者效果不佳[8]–[11]。因此对于严重威胁到患者生命的CRS且IL-6受体抑制剂以及糖皮质激素不能迅速控制的情况下，需要探索一种更高效的手段控制CRS。体外血液净化技术包括血浆置换、血液滤过、血液透析滤过（HDF）等，既往有分别应用血浆置换和血液滤过控制CAR-T治疗后CRS的个案报道[12]–[13]，结果显示患者CRS相关细胞因子明显下降，症状改善，无感染、出血、血栓等肾脏替代治疗的相关并发症；目前暂无HDF治疗CAR-T相关CRS的报道。本研究中，我们尝试使用HDF对CAR-T疗法引起的CRS进行治疗，探讨HDF治疗CAR-T细胞后3～4级CRS的安全性及有效性。

## 病例与方法

1. 病例资料：以2015年7月至2021年7月深圳大学第一附属医院/深圳市第二人民医院血液内科收治的CAR-T细胞治疗后发生3～4级CRS且接受HDF治疗的3例患者为研究对象，研究方案经深圳大学第一附属医院/深圳市第二人民医院医院伦理委员会批准（批件号：20140065），所有患者治疗前均已完全知情同意并签署同意书。例1为31岁男性，诊断急性B淋巴细胞白血病（B-ALL），复发1次后再进行3个疗程化疗，复查骨髓仍为未缓解（NR）（骨髓原始淋巴细胞85％）；例2为31岁女性B-ALL患者，复发2次，第2次复发后进行3个疗程化疗后，疗效评价为NR（骨髓原始淋巴细胞99％）；例3为49岁男性EB病毒（EBV）相关弥漫大B细胞淋巴瘤（DLBCL）患者，经历11个疗程化疗后病灶处于持续进展状态，淋巴结持续增大。CAR-T细胞治疗前3例患者的肝、肾、凝血功能均正常，生命体征平稳。

2. 预处理及CAR-T细胞输注过程：提前采集患者外周血单个核细胞外送至实验室制作第二代CAR细胞，接受以FC（氟达拉滨25～30 mg·m^−2^·d^−1^× 3 d，环磷酰胺250～300 mg·m^−2^·d^−1^×3 d）为基础的预处理方案，给予苯海拉明预防过敏反应、对乙酰氨基酚预防发热。CAR-T细胞的回输分3 d进行，分别回输总量的10％、30％、60％；记CAR-T细胞回输的第1天为d 0，回输的第2天为d 1，回输的第3天为d 2。例2因在d 1出现头晕、胸闷，血氧饱和度及血压下降，考虑CRS反应，取消了第3次CAR-T细胞的回输。例1、例2、例3 CAR-T细胞回输量分别为37.5×10^6^、21×10^6^、62×10^6^。

3. CRS的分级：根据美国移植和细胞治疗学会（ASTCT）制定的CRS分级指南进行CRS的分级。指南显示，CRS诊断的先决条件是发热（CAR-T细胞给药后的24 h至3周内体温≥38 °C），CRS等级和严重程度的主要决定因素是低血压和缺氧[14]–[15]。

4. 3～4级CRS的治疗：①IL-6抑制剂+糖皮质激素治疗：采用托珠单抗8 mg/kg（若效果不佳，第2天再应用1次）和甲泼尼龙80 mg/d×3 d进行治疗并进入重症监护室（ICU）管理。为防止病情进一步恶化和脏器损害，使用血管活性药物（如多巴胺）和（或）高流量吸氧（通过鼻插管、面罩）作为应急处理方案。密切监测心脏功能。例2使用血管活性药物和正压通气（持续正压气道、双水平正压气道、插管、机械通气）。②HDF治疗：患者IL-6≥5 000 ng/L或每天IL-6上升速度≥1 000 ng/L且患者出现严重并发症（如血管活性药物不能控制的休克、血氧持续下降、出现神经精神症状、脏器功能受损等）开始HDF治疗。HDF具体操作：在股静脉采用单针双腔静脉建立起血液通路，连接血液灌流机和血液通路，血流量控制为150 ml/min，治疗时间为20～50 h；治疗期间采用低分子肝素行常规抗凝处理，每8 h 1次。③对症支持治疗：纠正电解质紊乱、抗感染、升白细胞治疗等。

## 结果

1. 临床症状：3例患者分别在d 6、d 1、d 10发生3级、4级、3级CRS，主要症状为发热、低血压、血氧饱和度下降、胸闷、头晕/头痛等症状，体温热峰分别为40.0、40.8、39.4 °C，最低血压分别为80/47、83/42、82/40 mmHg（1 mmHg＝0.133 kPa），最低血氧饱和度（未吸氧状态下）分别为86％、84％、88％；未出现神经毒性相关症状。

2. 疗效评估：出现症状当日例1、例2予托珠单抗+甲泼尼龙，例3单用托珠单抗控制CRS，均未得到有效控制，且在次日再次应用托珠单抗±甲泼尼龙后，3例患者体温进一步升高，血压及血氧饱和度开始下降，3 d内IL-6迅速上升（图1），应用血管活性药物及吸氧治疗无法维持生命体征平稳，及时进入ICU管理并进行HDF治疗控制CRS，3例患者持续HDF时长分别为20、40、49 h，在使用HDF的过程中体温热峰开始下降，血压、血氧饱和度逐渐回升至正常，HDF治疗后12 h内脱离血管活性药物及吸氧治疗（表1）。

**图1:**
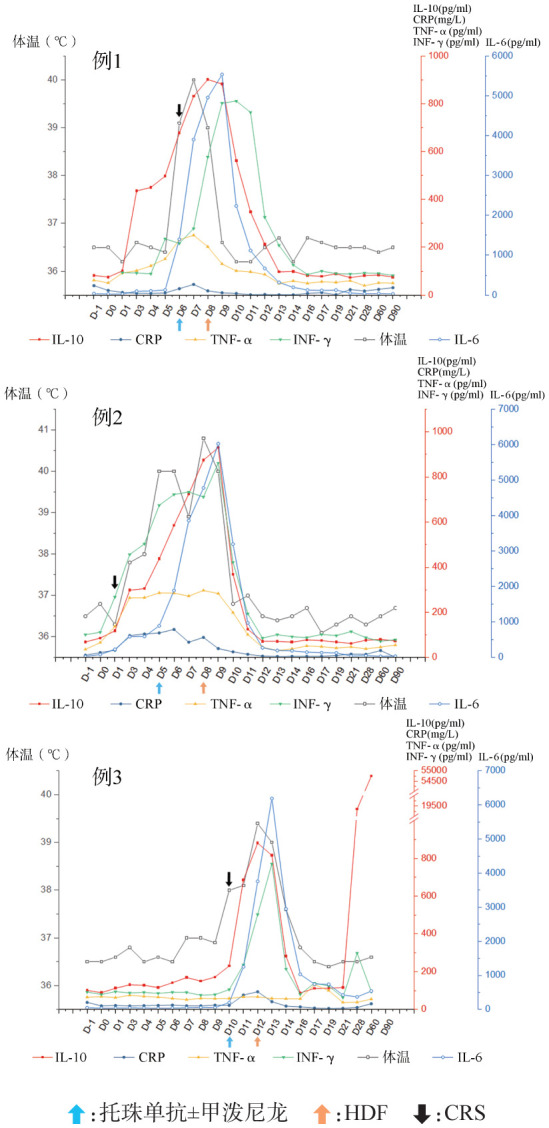
3例患者CAR-T细胞治疗过程中的体温及炎症因子水平变化 CAR-T细胞：嵌合抗原受体T细胞；CRS：细胞因子释放综合征；HDF：血液透析滤过

**表1:** 3例接受CAR-T细胞治疗血液肿瘤患者CRS治疗前及治疗后的临床症状变化

病例	发热	低血压	低血氧饱和度	胸闷	头晕/头痛	精神异常	行为异常	意识障碍
CRS初始症状								
例1	+	+	+	+	+	−	−	−
例2	+	+	+	+	+	−	−	−
例3	+	+	+	+	+	−	−	−
托珠单抗±甲泼尼龙治疗后								
例1	+	+	+	+	+	−	−	−
例2	+	+	+	+	+	−	−	−
例3	+	+	+	+	+	−	−	−
HDF后								
例1	−	−	−	−	−	−	−	−
例2	−	−	−	−	−	−	−	−
例3	−	−	−	−	−	−	−	−

注：CAR-T细胞：嵌合抗原受体T细胞；CRS：细胞因子释放综合征；HDF：血液透析滤过

3. 随访结果：HDF治疗后共随访3个月，均未出现HDF治疗相关不良反应（如出血、感染、内瘘狭窄、血栓等）。例1、例2在d 14、60、90完善骨髓细胞学检查提示未见原始淋巴细胞，骨髓流式微小残留病灶（MRD）阴性，疗效评估为完全缓解（CR）。例3在CAR-T细胞治疗后1个月因原发病进展，在CAR-T细胞治疗后2个月死亡。

4. CAR-T细胞拷贝数：3例患者CAR-T细胞拷贝数分别在d 2、2、7出现峰值，随后下降，并在d 21开始维持低水平，但仍可检测到CAR-T细胞存在体内；而例3在d 60时CAR-T细胞拷贝数明显升高，CAR-T细胞体内明显扩增的时间与患者原发病进展的时间相一致。

5. CRS实验室指标变化：3例患者在使用托珠单抗±甲泼尼龙后细胞因子（IL-6、IL-10、TNF-α、IFN-γ）未得到控制并仍在上升，其中以IL-6上升最为显著；体温热峰以及CRP同样上升。予以HDF治疗后细胞因子快速下降，CRP下降的速度较细胞因子缓慢，同时体温逐渐恢复正常，但例3细胞因子虽然下降但一直未恢复至正常值。例1、例2 HDF治疗后细胞因子均处于低水平状态；例3在CAR-T治疗后1个月原发病进展，细胞因子特别是IL-10在病情进展的同时出现了明显上升（图1）。

## 讨论

CRS相关的细胞因子和炎症标志物主要包括IL-6、IL-10、TNF-α、IFN-γ、IL-1、IL-2、IL-4、IL-8、可溶性IL-2Rα、C反应蛋白（CRP）、铁蛋白（FER）、颗粒酶B等[16]。其中，IL-6的升高被认为是症状的主要驱动因素之一，也是预测CRS的重要指标，更是发生CAR-T相关CRS的核心环节[17]–[18]，本研究中也证实患者出现CRS时以IL-6升高最明显。目前托珠单抗已被广泛用于治疗CRS并取得不俗的疗效[19]–[21]，但Le等[21]研究表明CTL019系列CAR-T细胞治疗后所致CRS的患者中69％的患者在使用托珠单抗后12 d内缓解，中位时间为4 d，而KTE-C19系列中53％的患者7 d内获得缓解，中位缓解时间为4.5（2～7）d，在患者病情进展迅速的情况下，不能快速控制；而大剂量使用糖皮质激素通常用于对IL-6受体抑制剂无效的难治性CRS[9],[22]。目前暂时没有相关文献比较托珠单抗单药、糖皮质激素单药和托珠单抗联合糖皮质激素哪种治疗效果及安全性更优，但是有研究表明糖皮质激素会减弱CAR-T细胞治疗的效果[22]。

本研究中我们尝试的HDF技术属于体外血液净化技术中血液透析和血液滤过的结合[23]–[24]，清除方式包括了弥散和对流，可以同时实现对小分子物质及中分子物质的清除[25]–[26]。血浆置换是体外血液净化技术中的另外一种方式，它不仅可以清除体内中、小分子的代谢毒素，还能清除蛋白、免疫复合物等大分子物质，可直接将患者血浆中的炎性物质置换出来[27]–[28]。体外血液净化技术的优点在于它能直接将细胞因子从体内清除，而不受限于某个单一细胞因子受体或信号传导；缺点在于需要建立血管通路，可能出现出血、感染、内瘘狭窄、血栓等风险。理论上讲，CRS相关细胞因子属于中分子，不论是HDF、血浆置换均有效，但由于异体新鲜血浆可能会带来过敏反应、血源性感染等风险，HDF相对会更安全；因此我中心目前更倾向于应用HDF治疗CRS。

目前应用体外净化技术治疗CAR-T后CRS的研究只有2例个案报道，其中一篇报道应用血液滤过能控制急性淋巴细胞白血病患者CAR-T治疗后严重的CRS反应，该患者最高IL-6达到10 000 ng/L，血液滤过后IL-6迅速下降，同时未发生体外血液净化技术相关不良反应[13]。另一个案报道显示，应用血浆置换治疗CAR-T相关CRS 1例，患者的CRS症状得到控制，体温及细胞因子在血浆置换结束后的当天恢复至正常水平[12]。此外，其他疾病（如全身炎症反应综合征、噬血细胞性淋巴组织细胞增多症、巨噬细胞活化综合征和COVID-19等）均可导致CRS，IL-6等细胞因子水平快速升高[29]，近期有文献报道应用血液滤过或血液透析控制COVID-19相关的CRS，患者体内细胞因子水平迅速降低，但并不能降低CRS相关死亡率[30]。

基于此，本研究中的3例患者在使用托珠单抗±甲泼尼龙后炎症指标及症状均未得到控制，且出现了进行性加重的情况，血管活性药物及吸氧均不能维持患者基本生命体征，我们及时应用HDF治疗1次后，细胞因子快速下降，同时体温恢复至正常，生命体征恢复平稳，且未出现HDF相关并发症；其中只有例3的IL-10在第28天陡然升高，这是由于例3为EBV相关的DLBCL，EBV的BCRFL编码框与人IL-10同源，在患者病情进展前期即会出现EBV激活且IL-10升高[31]。虽然HDF是有创操作，但并不是长期使用治疗，3例患者效果明显且安全性可。HDF治疗CRS时机也是比较重要的一环，我们应用托珠单抗±甲泼尼龙治疗后，仅在患者IL-6≥5 000 ng/L或每日IL-6上升速度≥1000 ng/L且患者出现严重并发症的情况下使用HDF。虽然从结果中我们看到HDF能够快速滤过患者体内细胞因子，但它是否对细胞因子持续升高的患者有明显效果，目前没有相关文献进行报道。HDF快速降低细胞因子是在CRS急性期采用的“特别手段”，对于IL-6抑制剂或糖皮质激素无效或耐药的CRS患者是不错的选择。

由于我院CAR-T细胞治疗后出现进展迅速的CRS患者病例数少，本次回顾性研究样本量较小，但至少可以说明，对于IL-6受体抑制剂治疗无效的3～4级CRS，HDF可以作为一种安全可行的方案，对提高3～4级CRS治疗效果、改善预后也有一定帮助。
